# Supplementary material for: Med23 serves as a gatekeeper of the myeloid potential of hematopoietic stem cells
Source: Nat Commun. 2018 Sep 14;9:3746. doi: 10.1038/s41467-018-06282-2 (PMC6138688; doi:10.1038/s41467-018-06282-2)
Supplement: Supplementary file 1 — Supplementary Information [file 41467_2018_6282_MOESM1_ESM.pdf]

Med23 serves as a gatekeeper of the myeloid potential of hematopoietic stem cells

Chen et al.

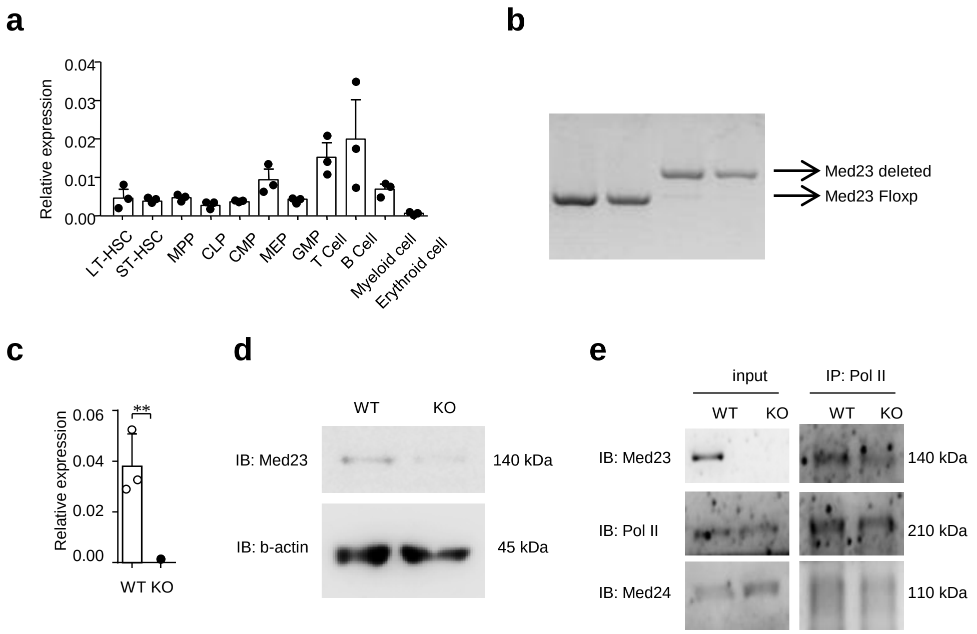

Supplementary Figure 1 Med23 expression in different hematopoietic cells and efficiency of knockout after poly(I:C) or tamoxifen administration.

(a) Med23 expression in different hematopoietic cells. LT-HSC: CD34$^-$CD135$^-$LSK(Lineage$^-$ cKit$^+$Sca1$^+$); ST-HSC: CD34$^+$CD135$^-$LSK; MPP: CD34$^+$CD135$^+$LSK; CLP: CD127$^+$cKit^{low} Sca1^{low} Lineage$^-$; CMP:CD34$^+$CD16/32$^-$LK (Lineage$^-$cKit$^+$Sca1$^-$); GMP:CD34$^+$CD16/32$^+$LK; MEP:CD34$^-$ CD16/32$^-$LK; T cell: CD3$^+$ cell; B cell: B220$^+$ cell; Myeloid cell: Gr1/Mac1$^+$ cell; erythroid cell: Ter119$^+$ cell. Normalized to Gapdh, n=3.

(b) Representative PCR analysis of genomic DNA extracted from bone marrow cells of WT and KO mice at 21 day after poly(I:C) administration.

(c) Relative expression level of Med23 in sorted WT and KO HSCs, normalized to Gapdh (n = 3 for each genotype).

(d) Western blotting analysis of Med23 protein in Lineage$^-$cKit$^+$ cells of WT and KO mice after tamoxifen administration

(e) Western blotting analysis of Med23 and Med24 protein through immunoprecipitation with Pol II antibody in hematopoietic progenitor cells (Lineage$^-$cKit$^+$ population).

The data are means ± S.D., for all panels: **$p < 0.01$ by Student's t-test.

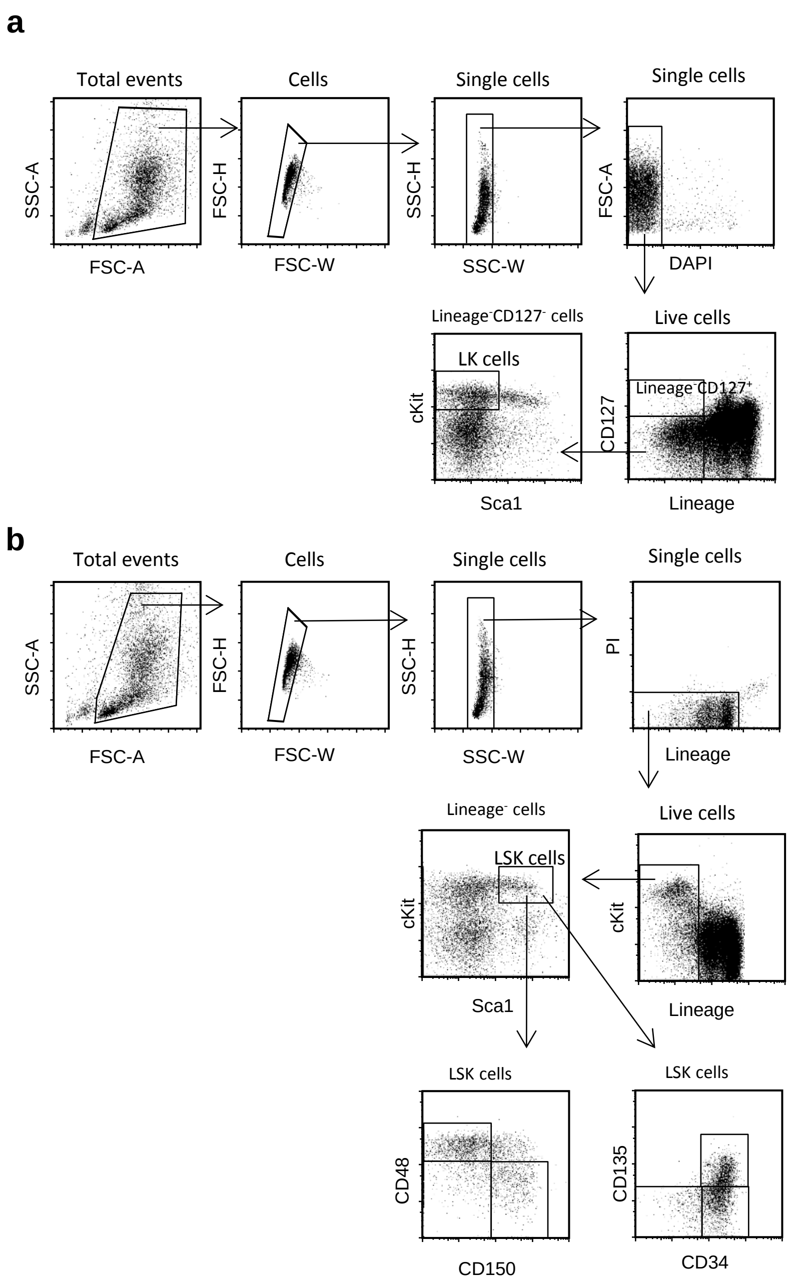

Supplementary Figure 2 Gating strategies used for gating or sorting.
(a) Gating strategy for HPC cells presented on Fig. 2b,c,d and e. (b) Gating strategy for HSC cells presented on Fig. 3.

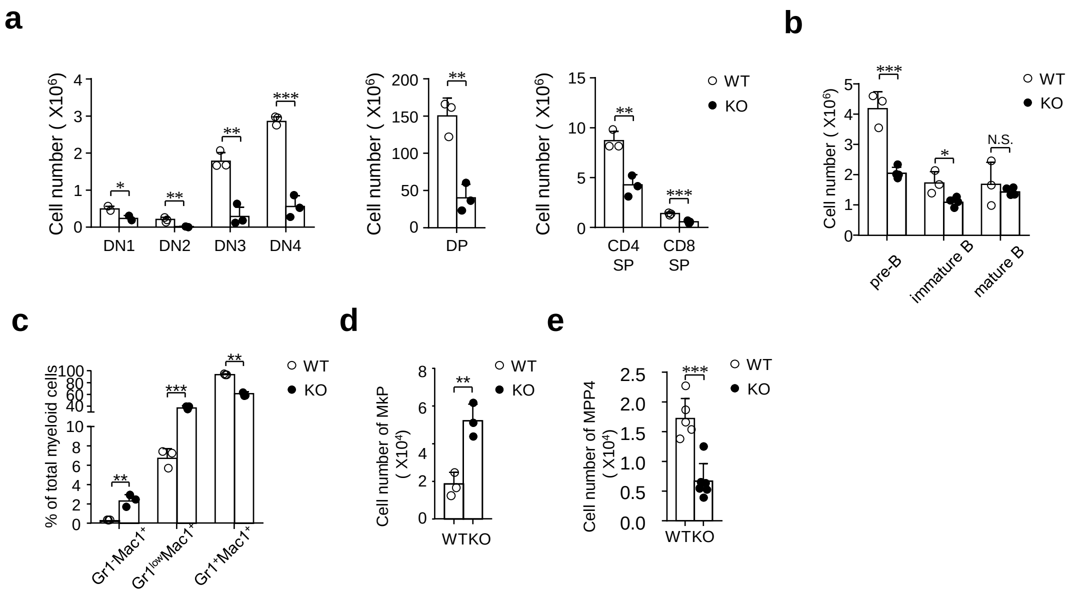

Supplementary Figure 3 Abnormal T cell and B cell development and increased Mk progenitors in KO mice.

(a) Absolute cell number of T cells at different stages during T cell development in WT and KO thymuses (*n*=3). DN, double negative; DP, double positive; SP, single positive.

(b) Absolute cell number of B cells at different stages during B cell development in WT and KO bone marrows (WT, *n*=3; KO, *n*=4).

(c) Percentages of neutrophil cells ($Gr1^+Mac1^+$), immature myeloid cells. ($Gr1^{low}Mac1^+$) and other myeloid cells ($Gr1^-Mac1^+$) in myeloid lineages ($GR1^+/Mac1^+$)(*n*=3).

(d) Absolute cell number of Mk progenitor cells ($CD41^+CD150^+LS^-K$, MkP) in bone marrows of WT and KO mice (*n*=3).

(e) Absolute cell number of MPP4 cells ($CD34^+CD135^+CD150^-CD48^+LSK$) in bone marrows of WT and KO mice (WT, *n*=5; KO, *n*=6).

The data are means ± S.D., for all panels: N.S.: no significance. *$p < 0.05$; **$p < 0.01$; ***$p < 0.001$ by Student's *t*-test.

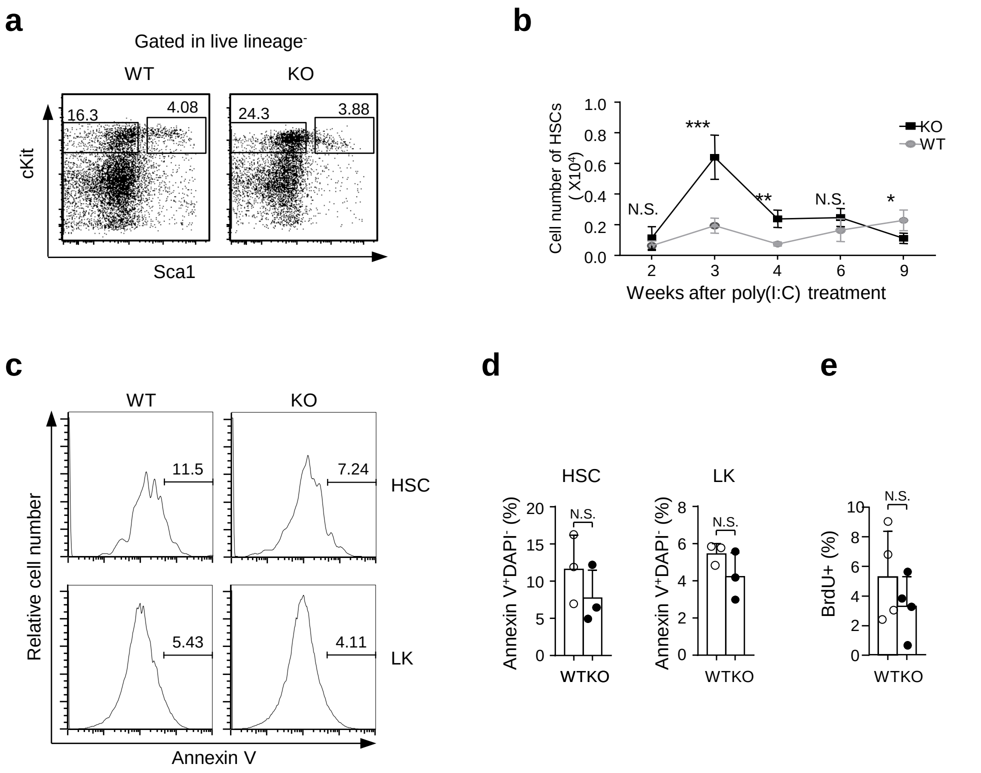

Supplementary Figure 4 The number of HSCs after loss of Med23 and normal survival and proliferation in Med23-deficient HSCs.

(a) Representative FACS dot plots of WT and Med23-deficient bone marrow after administration of poly(I:C). Lineage$^-$ viable cells are shown.

(b) Absolute cell number of HSCs (CD34$^-$CD150$^+$CD48$^-$CD135$^-$LSK) in bone marrows from WT and KO mice at indicated time points post poly (I:C) administration.

(c and d) Representative histograms of the Annexin V staining profile (c) and the percentage of HSCs (d) and LKs from WT and KO mice that undergo apoptosis (Annexin V$^+$ DAPI$^-$) (*n*=3).

(e) Percentages of BrdU incorporated HSCs in WT and KO mice at 18-hour post BrdU injection.

The data are means ± S.D., for all panels: N.S.: no significance. $*p < 0.05$; $**p < 0.01$; $***p < 0.001$ by Student's *t*-test.

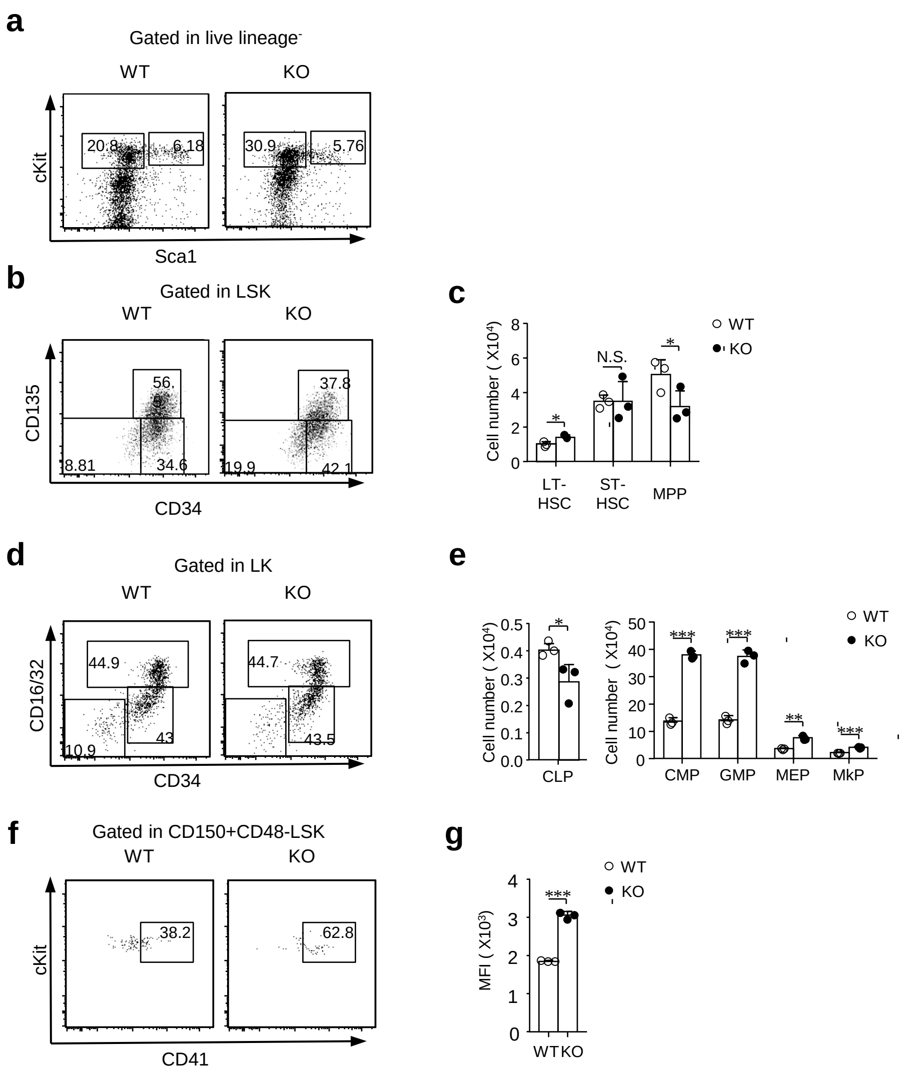

Supplementary Figure 5 UBC-cre/ERT2; Med23$^{fl/fl}$ mice phenocopy Mx1-Cre; Med23$^{fl/fl}$ mice.
(a) Representative FACS plots of WT and Med23-deficient bone marrow at 21-day post tamoxifen administration. Lineage- viable cells are shown.
(b and c) Representative dot plots (b) and absolute cell numbers (c) of LT-HSC (CD34^{-}CD135^{-} LSK), ST-HSC (CD34^{+}CD135^{-} LSK) and MPP (CD34^{+}CD135^{+} LSK) from tamoxifen-induced WT and KO mice (WT, n=3; KO, n=3). LSK populations are shown.
(d and e) Representative dot plots (d) and absolute cell numbers (e) of MEP(CD34^{-}CD16/32^{-} LSK), ST-HSC (CD34^{+}CD16/32^{-} LSK) and MPP (CD34^{+}CD16/32^{+} LSK) from tamoxifen-induced WT and KO mice (WT, n=3; KO, n=3). LK populations are shown.
(f) Representative dot plots showing CD41 expression within HSCs in WT and KO mice induced by tamoxifen (n=3). HSC(CD150^{+}CD48^{-}LSK) population are shown.
(g) Mean fluorescence intensity (MFI) of CD41 expression in HSCs in WT and KO mice induced by tamoxifen (n=3).
The data are means ± S.D., for all panels: N.S.: no significance. *$p < 0.05$; **$p < 0.01$; ***$p < 0.001$ by Student's t-test.

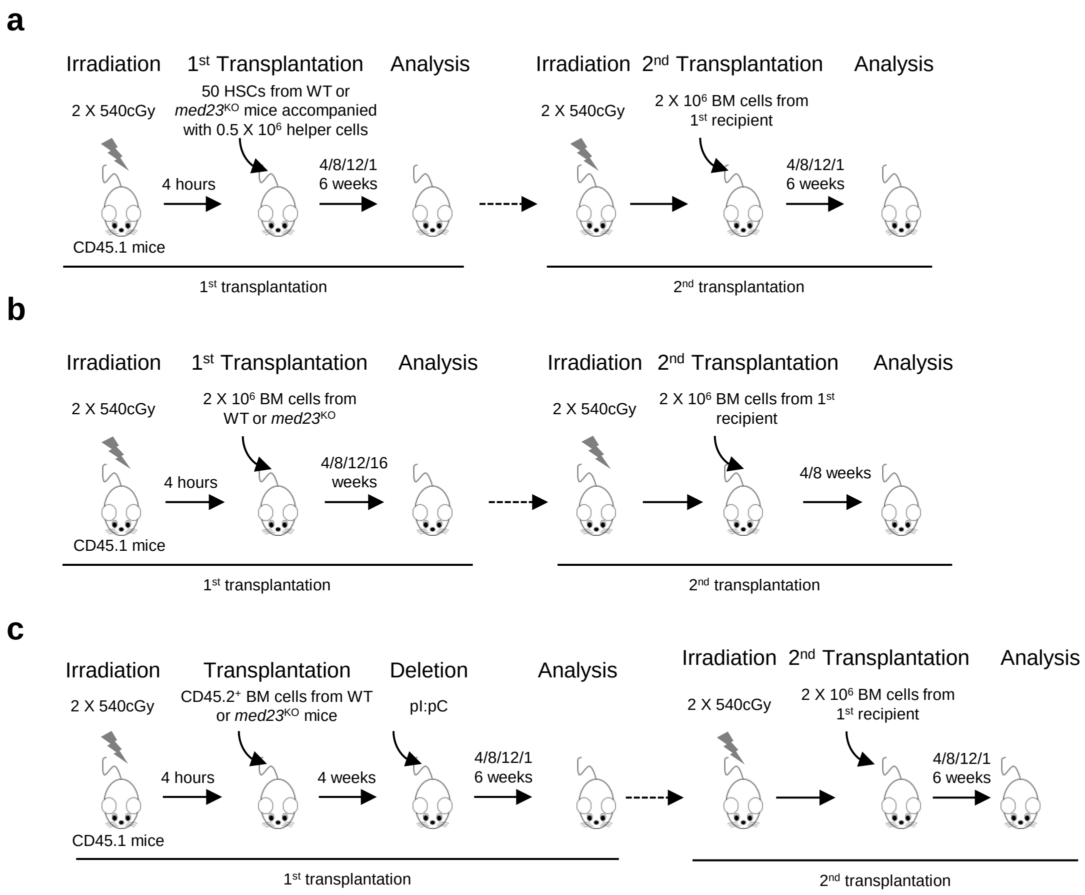

Supplementary Figure 6 Experimental design for bone marrow transplantation.
(a) Recipients were transplanted with 50 HSCs (CD45.2) from WT or KO mice, along with 500,000 bone marrow cells (CD45.1). Donor chimerism of PBMC was analyzed every 4 weeks post-transplantation. At 16 week after first transplantation, recipient mice were sacrificed and the chimerim of donor HSCs was analyzed. Then 2,000,000 bone morrow cells from primary recipients were transplanted into secondary recipients. Donor chimerism of PBMC in secondary recipients was analyzed every 4 weeks post-transplantation.
(b) Primary recipients were transplanted with 2,000,000 total bone marrow cells (CD45.2) from WT and KO mice. Donor chimerism of PBMC was analyzed every 4 weeks post-transplantation. Secondary recipients were transplanted with 2,000,000 bone morrow cells from primary recipients. Donor chimerism of PBMC in secondary recipients was analyzed every 4 weeks post-transplantation. Animal survival was record after secondary transplantation.
(c) 1,000,000 bone marrow cells isolated from WT or KO mice were mixed with equal numbers of CD45.1 WT competitors then cells were transplanted into lethally irradiated recipient mice. poly(I:C) administration was done to induce Med23 deletion at 4 weeks after transplantation. Donor chimerism of PBMC was analyzed every 4 weeks post-transplantation.

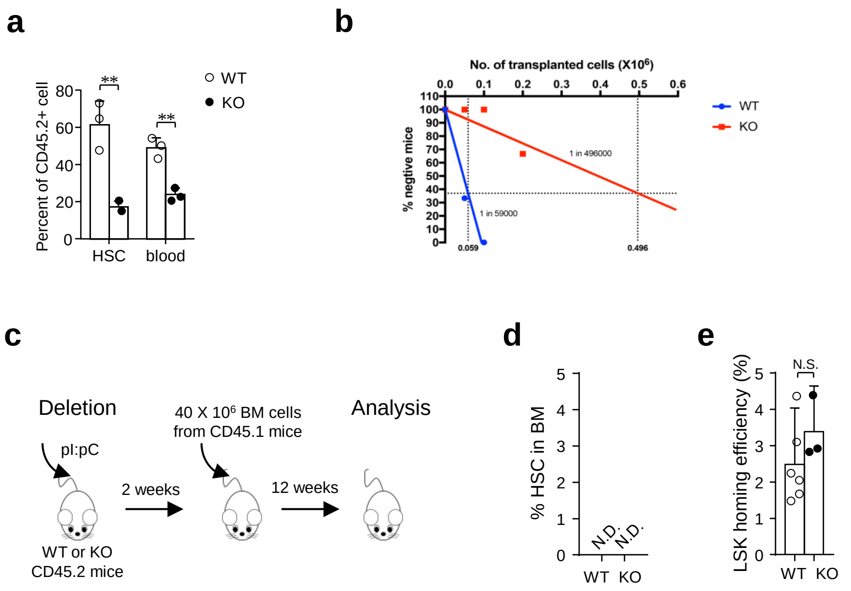

Supplementary Figure 7 Impaired self-renewal but normal niche retention and homing capacity of Med23-deficient HSCs.

(a) Donor chimerism of HSC (CD150+LSK) and PBMC in primary recipients transplanted with 1,000,000 bone marrow cells (CD45.2) of WT and KO mice, along with 1,000,000 bone marrow cells (CD45.1) followed by poly(I:C) administration at 12 week post-transplantation. Donor chimerism was analyzed at 6 weeks after administration (*n*=3).

(b) Frequency analysis (top) for WT and KO HSCs, determined by Poisson statistics, at 12 weeks; P0 = 59000 (WT) and P0 = 496000(KO). Data represent one experiment; *n* = 3 mice/group.

(c) Experimental design for HSC bone marrow retention assay. Non-irradiated WT and KO recipient mice (CD45.2) were transplanted with 40,000,000 total bone marrow cells (CD45.1) . Chimerism of donor HSCs (CD45.1) was performed at 12 weeks post-transplantation.

(d) Percentages of HSC (CD45.1) in the bone marrow from WT and KO mice (*n*=4).

(e) Analysis of the homing capacity of HSCs (LSKs) from WT and KO mice. 10,000 sorted LSKs from WT and KO mice were labeled with CSFE then transplanted into the recipient mice. 16 hours later, the recipients were sacrificed and the percentages of CFSE+LSK in the bone marrows were analyzed (WT, *n*=6, KO, *n*=3).

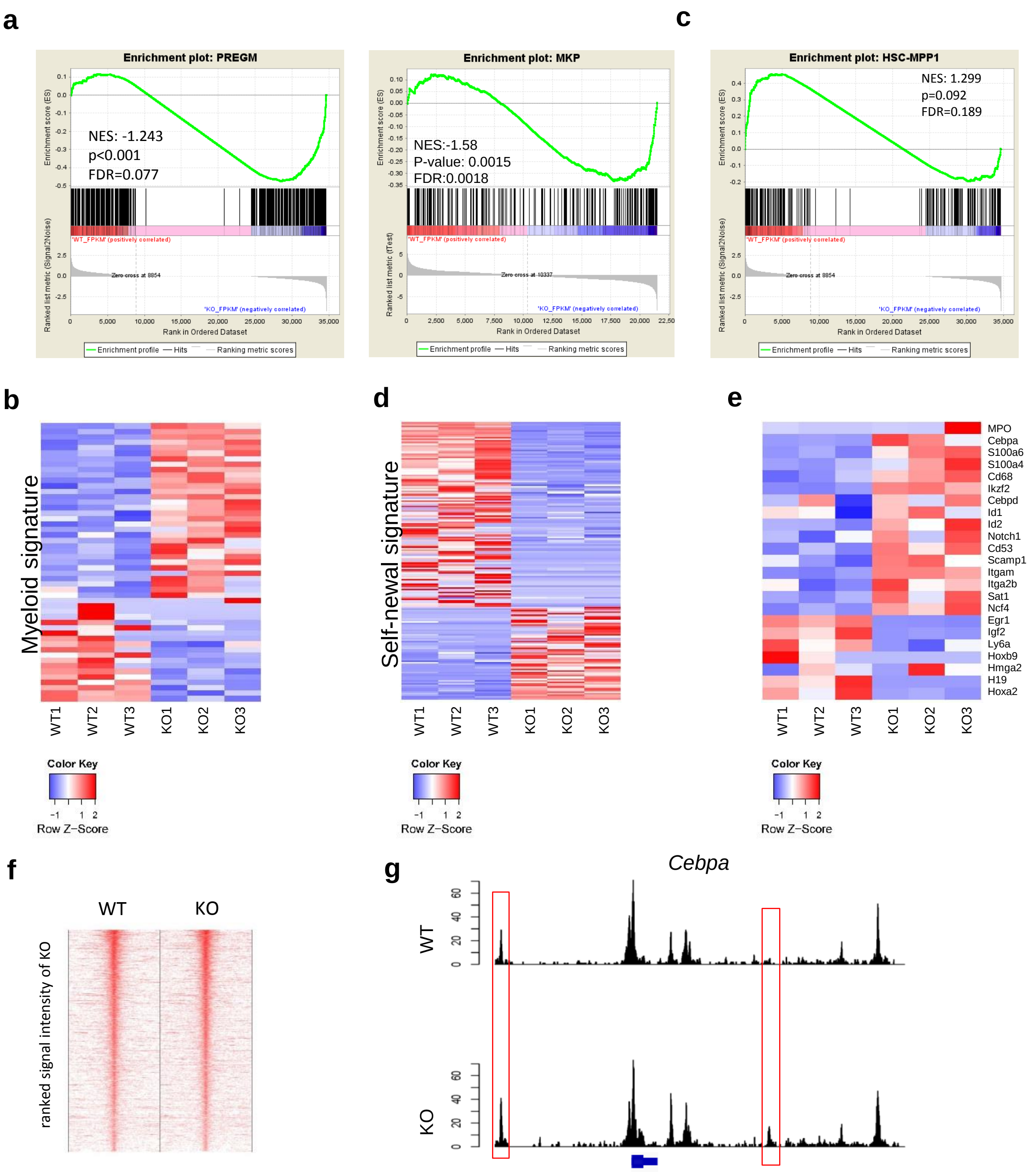

Supplementary Figure 8 Med23-deficient HSCs mimic myeloid-primed HSCs

(a) Gene-set enrichment analysis of myeloid (left) and MkP (right) signature genes comparing WT with KO HSCs (*n*=3 for each genotype).

(b) Heatmap of differentially expressed myeloid signature genes between WT and KO HSCs.

(c) Gene-set enrichment analysis of self-renewal signature genes comparing WT with KO HSCs (*n*=3 for each genotype).

(d) Heatmap of differentially expressed self-renewal signature genes between WT and KO HSCs.

(e) Expression of representative genes with known functions in stem cell maintenance and myeloid cell development.

(f) Genome-wide analysis of chromatin status of gene transcription start site (TSS) using ATAC-Seq.

(g) Representative tracks showing the loci of the key myeloid gene, *Cebpa*. Blue region shows the locus of *Cebpa* gene .

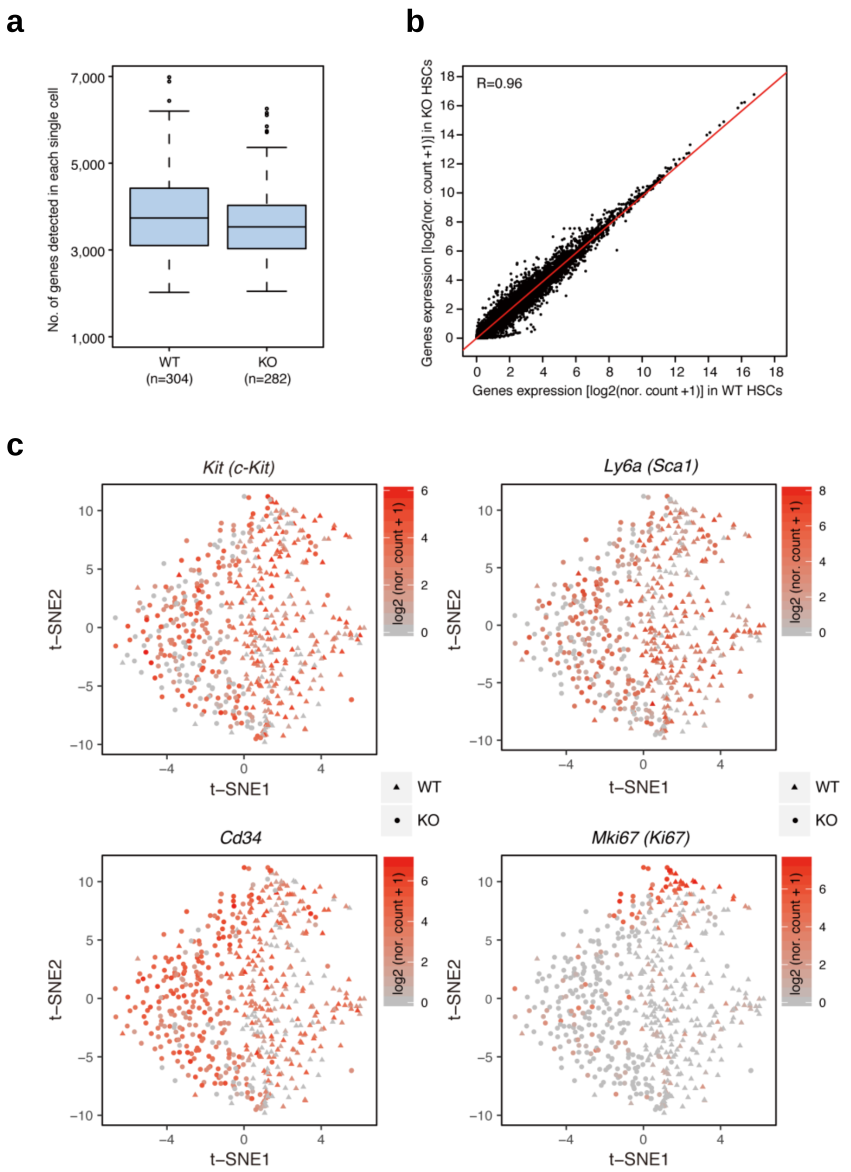

Supplementary Figure 9 The single cell transcriptome of Med23-deficient HSCs is generally similar to that of WT HSCs.

(a) Box plots of the number of genes detected in each single cell (WT, n = 304; KO, n = 282). The lines indicate the median.

(b) The correlation of genes expression between WT and KO HSCs. “R” indicates the Spearman’s correlation coefficient.

(c) Expression of representative genes exhibited on the t-SNE plots. *Kit* (also known as *c-Kit*), *Ly6a* (also known as *Sca1*) and *Cd34* are known marker genes expressed in HSCs. *Mki67* (also known as *Ki67*) marks the HSCs arrest in G0- (not expressed) or G1- (expressed) phase of the cell cycle. Normalized counts of each gene in single cells were used.

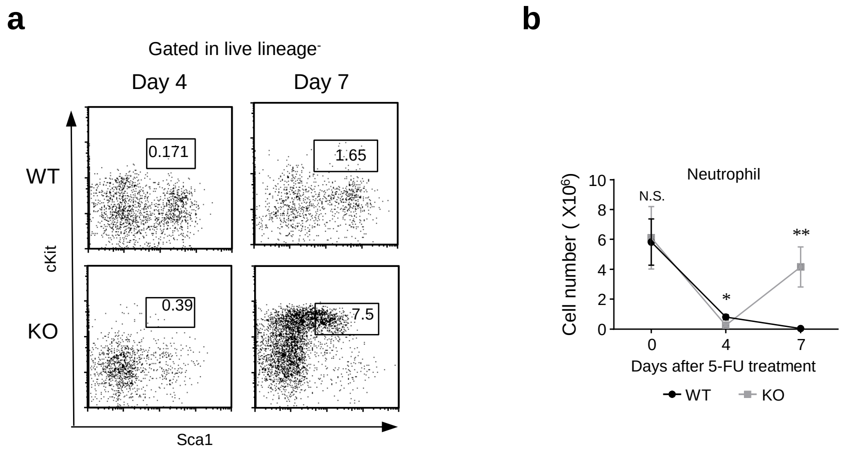

Supplementary Figure 10 Med23-deficient mice show better recovery upon 5-FU treatment.

(a) Representative dot plots of LSKs from WT and Med23-deficient mice at day 4 and day 7 after single 5-FU injection. Lineage$^-$ viable cells are shown.

(b) Absolute cell number of neutrophil cells (Gr1$^+$Mac1$^+$)) from WT and Med23-deficient mice at different time points after single 5-FU injection (*n*=3).

Supplementary Table 1 Primer pairs for genotyping and qPCR

Primer name	Sequence	use
Med23-GT-F	5′ -GCGGCCGCTATATGCACTGTTAGTGATT-3′	genotyping
Med23-GT-R	5′ -GTCGACCTTAGAAGAAAGCTCAAACAT-3′	genotyping
Mx1-GT-F	5′ -CTCTGCTGCCTCCTGGCTTCT-3′	genotyping
Mx1-GT-R	5′ -CGAGGCGGATCACAAGCAATA-3′	genotyping
UBC-CreERT2-F	5′ -GACCAGGTTCGTTCACTCA-3′	genotyping
UBC-CreERT2-R	5′ -CAAGTTAGGAGCAAACAGTAGC-3′	genotyping
Med23-KO-1	5′ -GGATGAACGGTGGAAAGACCTT-3′	genotyping
Med23-KO-2	5′ -GGCTATGACCTGTAGATGCTGAGT-3′	genotyping
Med23-KO-3	5′ -GCCTGAAGGTATCCACAAAGTCT-3′	genotyping
Med23-RT-F	5′ -GCCTATTGCCGGCCTACTTT-3′	qPCR
Med23-RT-R	5′ -CTGTGGGCCTGAAGGTATCC-3′	qPCR
Gapdh-RT-F	5′ -TGGTGAAGGTCGGTGTGAACGG-3′	qPCR
Gapdh-RT-R	5′ -ACTGTGCCG TTGAATTTGCCG-3′	qPCR
