# Supplementary material for: Med23 serves as a gatekeeper of the myeloid potential of hematopoietic stem cells
Source: Nat Commun. 2018 Sep 14;9:3746. doi: 10.1038/s41467-018-06282-2 (PMC6138688; doi:10.1038/s41467-018-06282-2)
Supplement: Supplementary file 3 — Description of Additional Supplementary Files [file 41467_2018_6282_MOESM3_ESM.pdf]

Legends for Supplementary Data files

Supplementary Data 1. Differentially expressed genes between WT and Med23-deficient HSCs by single cell RNA-seq analysis.

Supplementary Data 2. Detailed information for antibodies and reagents.

Supplementary Data 3. Oligo-dT primers for single cell RNA-seq library constructions.
